# Urinary C-peptide creatinine ratio as a non-invasive diagnostic tool for differentiating type 1 from type 2 diabetes mellitus in adult Emirati population: a prospective validation study

**DOI:** 10.3389/fendo.2025.1687920

**Published:** 2025-10-16

**Authors:** Fayez Alshamsi, Afrin Pathan, Javed Yasin, Charu Sharma, Hussain Abdalla Alshemsi, Abdelrahman Alblooshi, Mohamed Abdulkareem AlAwadhi, Ahmad Abdulrazak Alali, Maitha Alkuwaiti, Bachar Afandi, Juma AlKaabi, Adnan Agha

**Affiliations:** ^1^ Department of Internal Medicine, College of Medicine and Health Sciences, United Arab Emirates University, Al Ain, United Arab Emirates; ^2^ Division of Endocrinology, Department of Internal Medicine, Tawam Hospital, Al Ain, United Arab Emirates

**Keywords:** urinary C-peptide creatinine ratio, type 1 diabetes mellitus, type 2 diabetes mellitus, diagnostic accuracy, beta-cell function, biomarker, cost-effectiveness

## Abstract

**Aims:**

The precise differentiation between Type 1 diabetes mellitus (T1DM) and Type 2 diabetes mellitus (T2DM) can be challenging in clinical practice, particularly in adults. We aimed to validate the diagnostic accuracy and performance of urinary C-peptide creatinine ratio (UCPCR) for distinguishing T1DM from T2DM in the Emirati population.

**Methods:**

This prospective cross-sectional study included 79 patients with diabetes (19 T1DM, 60 T2DM) from Tawam Hospital Diabetes Center, UAE. Post-prandial urine samples were collected for UCPCR measurement using chemiluminescent immunoassay. Receiver operating characteristic (ROC) analysis determined optimal cut-offs. Multivariable logistic regression and cost-comparison analyses were performed.

**Results:**

Mean UCPCR was significantly lower in T1DM compared to T2DM (0.29 ± 0.64 vs 1.44 ± 1.82 nmol/mmol, p<0.001). ROC analysis revealed that a UCPCR cut-off of <0.25 nmol/mmol achieved 100% sensitivity and 91.7% specificity for T1DM diagnosis (AUC 0.991, 95% CI: 0.978-1.000). In patients with diabetes duration <5 years, UCPCR maintained excellent discrimination (AUC 0.988, sensitivity 100%, specificity 91.7%). However, specificity declined in patients with a diabetes duration of >10 years (82.4%), with 15% of these longstanding T2DM patients exhibiting UCPCR values <0.25 nmol/mmol, reflecting progressive beta-cell decline. Multivariable regression identified UCPCR (OR 0.001; 95% CI: 0.000–0.012; p<0.001) as the strongest independent predictor of T1DM. Cost-comparison analysis demonstrated ≥ 90% cost reduction when compared with serum C-peptide or autoantibody panels.

**Conclusions:**

UCPCR < 0.25 nmol/mmol accurately identifies T1DM in the Emirati population. This cost-effective, non-invasive test could improve clinical practice through enhanced diagnostic accuracy and reduced healthcare costs.

## Graphical Abstract

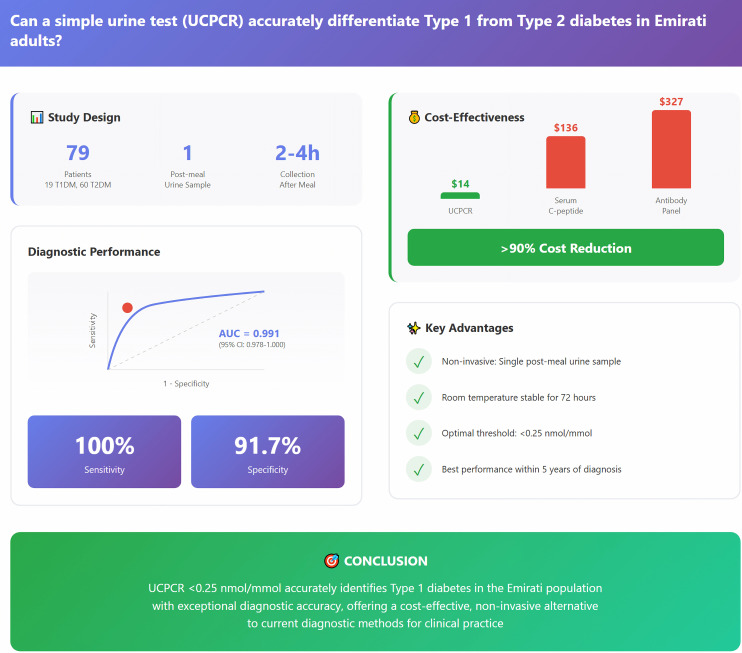

## Introduction

The correct classification of diabetes mellitus into its primary subtypes is a critical challenge for diabetologists, directly influencing therapeutic strategies, prognostic assessments, and long-term patient outcomes (1). Type 1 diabetes mellitus (T1DM) results from autoimmune-mediated destruction of pancreatic beta-cells leading to absolute insulin deficiency, while Type 2 diabetes mellitus (T2DM) arises from a complex interplay of insulin resistance and progressive beta-cell dysfunction (2). Despite these distinct pathophysiological mechanisms, clinical differentiation can be challenging in a significant number of patients, particularly in adults where traditional phenotypic distinctions may overlap (3).

The clinical implications of misclassifying the subtype of diabetes can extend far beyond academic interest. Patients with unrecognized T1DM may receive inappropriate oral hypoglycemic agents or glucagon-like peptide-1 (GLP-1) receptor agonists, potentially accelerating residual beta-cell destruction and precipitating diabetic ketoacidosis (4). Conversely, T2DM patients with severe insulin deficiency may experience prolonged periods of suboptimal glycemic control if their underlying pathophysiology remains unrecognized. Recent large-scale studies suggest that up to 35% of adults diagnosed with apparent T2DM may harbor T1DM or latent autoimmune diabetes in adults (LADA), underscoring the magnitude of this diagnostic challenge (5).

The complexity of diabetes classification has been further complicated by the recognition of intermediate phenotypes. LADA represents a slowly progressive form of autoimmune diabetes that often presents with clinical features indistinguishable from T2DM at diagnosis (6). Additionally, maturity-onset diabetes of the young (MODY) and ketosis-prone diabetes add further layers of complexity to the diagnostic landscape. These atypical presentations highlight the limitations of clinical criteria alone in accurately classifying diabetes subtypes.

C-peptide measurement has emerged as the definitive biomarker for assessing endogenous insulin secretion and, by extension, residual beta-cell function (7). This 31-amino acid peptide, cleaved from proinsulin during insulin biosynthesis, offers several analytical advantages over direct insulin measurement. Co-secreted with insulin in equimolar amounts, C-peptide exhibits minimal hepatic extraction (5-10% compared to 50-80% for insulin), resulting in more stable peripheral concentrations (8). Furthermore, its longer half-life (20–30 minutes versus 3–5 minutes for insulin) and lack of cross-reactivity with exogenous insulin make it an ideal marker for assessing endogenous insulin secretion in insulin-treated patients.

While the mixed-meal tolerance test (MMTT) with stimulated serum C-peptide measurement represents the current gold standard for beta-cell function assessment, its implementation faces significant practical barriers (9). The test requires fasting, standardized meal ingestion, multiple blood draws over 90–120 minutes, and specialized laboratory facilities. These requirements limit its utility in routine clinical practice, particularly in resource-constrained settings.

The urinary C-peptide creatinine ratio (UCPCR) has emerged as a promising alternative that addresses many limitations of serum-based testing. By capitalizing on the renal clearance of C-peptide and correcting for urinary concentration through creatinine normalization, UCPCR provides a stable, reproducible measure of integrated insulin secretion (10). The test offers numerous practical advantages: non-invasive collection suitable for home sampling; stability at room temperature for up to 72 hours with boric acid preservation; elimination of fasting requirements; and correlation with gold-standard MMTT results.

European validation studies have established UCPCR thresholds for severe insulin deficiency, with values <0.2 nmol/mmol demonstrating high sensitivity and specificity for T1DM diagnosis (11). However, emerging evidence suggests that diabetes pathophysiology exhibits significant ethnic variation, necessitating population-specific validation of diagnostic thresholds (12). For instance, East Asian populations often develop T2DM at a lower BMI and with greater visceral adiposity compared with European cohorts, reflecting fundamental differences in insulin resistance and beta-cell function that directly impact C-peptide interpretation. The Emirati population which faces one of the world’s highest diabetes burdens; with prevalence exceeding 15% in the adult population (13);presents a similar unique metabolic profile, making the application of European-derived diagnostic thresholds scientifically inappropriate and clinically risky.

The United Arab Emirates faces one of the world’s highest diabetes burdens, with prevalence exceeding 15% in the adult population (13). This epidemic encompasses both T1DM and T2DM, with unique epidemiological features including early age at onset, high rates of consanguinity potentially influencing monogenic diabetes prevalence, and rapid lifestyle transitions contributing to obesity and insulin resistance. Despite this substantial disease burden, population-specific diagnostic tools have not been validated for the Emirati population.

This study aimed to validate the diagnostic performance of UCPCR for differentiating T1DM from T2DM in the adult Emirati population, to determine optimal population-specific cut-off value, and to evaluate the cost-comparison of UCPCR implementation in routine clinical practice.

## Materials and methods

### Study design and setting

This prospective cross-sectional validation study was conducted at Tawam Hospital Diabetes Center, Al Ain, UAE, between October and December 2024. Tawam Hospital serves as a tertiary referral center for the Al Ain region, managing over 5,000 patients with diabetes annually. The diabetes center provides comprehensive care including specialist endocrinology services, diabetes education, and advanced diagnostic capabilities. The study protocol received approval from the Tawam Hospital Research and Ethics Committee (Approval Number MF2058-2023-988) and was conducted in accordance with the Declaration of Helsinki principles. All participants provided written informed consent, with parental consent and patient assent obtained for participants aged 12–17 years.

### Study participants

Consecutive patients attending diabetes clinics were screened for eligibility using a systematic approach where all eligible patients presenting during the study period (October-December 2024) were invited to participate, regardless of demographic characteristics. To minimize selection bias, we recruited from both morning and afternoon clinics across all weekdays, capturing diverse patient populations including employed individuals and retirees. The 1:3 ratio of T1DM to T2DM participants reflects the natural prevalence in our tertiary diabetes center population, where T1DM comprises approximately 20-25% of the adult diabetes cohort. No matching for age, gender, or BMI was performed to maintain real-world applicability. Potential referral bias was acknowledged as our tertiary center may see more complex cases; however, this enhances generalizability to similar specialized diabetes centers. Inclusion criteria comprised: (1) Emirati nationals aged ≥12 years; (2) established diagnosis of T1DM or T2DM for ≥6 months based on American Diabetes Association criteria (14); (3) ability to provide informed consent. The criterion of a minimum six-month diabetes duration was chosen to ensure metabolic stability and to exclude patients with transient hyperglycemia (e.g., stress-induced or medications like steroids related).

Diabetes classification was determined through comprehensive clinical assessment by experienced diabetologists incorporating multiple parameters: age at diagnosis, clinical presentation (presence of diabetic ketoacidosis, osmotic symptoms, weight loss), family history of diabetes, body mass index (BMI), insulin dependency from diagnosis, and available autoantibody results (when performed for clinical indications). T1DM diagnosis required absolute insulin dependence within six months of diagnosis with clinical features consistent with autoimmune diabetes. T2DM diagnosis required absence of absolute insulin dependence at diagnosis with clinical features suggesting insulin resistance.

Exclusion criteria included: (1) gestational diabetes or diabetes secondary to other conditions (pancreatitis, Cushing’s syndrome, medication-induced); (2) estimated glomerular filtration rate (eGFR) <30 mL/min/1.73m² to avoid confounding from impaired renal C-peptide clearance; (3) active urinary tract infection potentially affecting urinary protein excretion; (4) inability to provide informed consent; (5) suspected monogenic diabetes based on clinical criteria.

### Clinical data collection

Standardized case report forms captured comprehensive clinical data including demographic information, detailed diabetes history, anthropometric measurements, current medications, and diabetes complications. Height and weight were measured using calibrated equipment with BMI calculated as weight (kg)/height (m²). Blood pressure was measured after 5 minutes rest using automated sphygmomanometers. Laboratory data including glycated hemoglobin (HbA1c), lipid profile, and renal function were extracted from electronic medical records, with all tests performed within three months of UCPCR measurement.

### UCPCR sample collection and analysis

In accordance with established protocols for optimal C-peptide stimulation, post-prandial urine samples were collected 2–4 hours after a standardized mixed meal containing approximately 50–60 g of carbohydrates (15). Participants were instructed to empty their bladder before the meal and collect the subsequent void into sterile containers with boric acid preservative. This timing captures peak post-prandial C-peptide excretion while remaining practical for clinical implementation.

Samples were transported to the laboratory within 24 hours. The use of boric acid as a preservative is a key component of the protocol, as it ensures sample stability for up to 72 hours at room temperature, a critical feature for clinical utility and sample transport (16). Urinary C-peptide was measured using electrochemiluminescent immunoassay on the Cobas e411 platform (Roche Diagnostics, Basel, Switzerland), with analytical range 0.003-13.3 nmol/L and inter-assay coefficient of variation <5% (17). Urinary creatinine was measured using the kinetic Jaffe method with standardization traceable to isotope dilution mass spectrometry (18). UCPCR was calculated as urinary C-peptide concentration (nmol/L) divided by urinary creatinine concentration (mmol/L), expressing results in nmol/mmol (16).

### Statistical analysis

Statistical analyses were performed using SPSS version 29.0 (IBM Corp., Armonk, NY, USA). Sample size calculations were based on previous UCPCR validation studies reporting area under the curve (AUC) values of 0.85-0.95 for T1DM diagnosis (11). We calculated that 24 participants (12 per group) would provide 80% power to detect an AUC of 0.90 versus the null hypothesis of 0.50, with two-sided alpha 0.05. Our recruited sample of 79 participants (19 T1DM, 60 T2DM) exceeded this minimum requirement. However, the modest sample size for the T1DM group (n=19) necessitates caution in interpreting results where this group is the primary focus, such as sensitivity and positive predictive value, which are accompanied by wide confidence intervals.

Continuous variables were assessed for normality using Shapiro-Wilk test. Normally distributed variables were expressed as mean ± standard deviation and compared using independent t-tests. Non-normally distributed variables were expressed as median (interquartile range) and compared using Mann-Whitney U tests. Categorical variables were expressed as frequencies (percentages) and compared using chi-square or Fisher’s exact tests as appropriate.

Receiver operating characteristic (ROC) analysis determined UCPCR diagnostic performance for T1DM identification. The optimal cut-off was determined using Youden index (sensitivity + specificity - 1), balancing false positive and false negative rates. Diagnostic performance metrics included sensitivity, specificity, positive predictive value (PPV), negative predictive value (NPV), and diagnostic accuracy with exact binomial 95% confidence intervals.

Multivariable logistic regression analysis identified independent predictors of T1DM. Variables with p<0.10 in univariable analysis were entered into the multivariable model using forward stepwise selection. Model fit was assessed using Hosmer-Lemeshow goodness-of-fit test. Multicollinearity was evaluated using variance inflation factors.

Subgroup analyses examined UCPCR performance stratified by diabetes duration (<5 years, 5–10 years, >10 years), age groups, and BMI categories. Cost-comparison analysis compared UCPCR with current diagnostic strategies, incorporating test costs, diagnostic accuracy, and projected clinical outcomes. All statistical tests were two-sided with p<0.05 considered significant.

## Results

### Study population characteristics

Between October and December 2024, 92 patients were screened for eligibility. Thirteen patients were excluded: Five patients with eGFR <30 mL/min/1.73m², four patients with suspected monogenic diabetes, three were unable to provide consent, and one with secondary diabetes. The final analysis included 79 participants: 19 with T1DM and 60 with T2DM.

Baseline characteristics differed significantly between groups (**Table 1**). Patients with T1DM were younger at study enrollment (28.4 ± 16.2 vs 55.8 ± 15.3 years, p<0.001) and at diabetes diagnosis (17.8 ± 12.1 vs 42.3 ± 12.8 years, p<0.001). The T1DM group had lower BMI (26.2 ± 4.8 vs 30.0 ± 5.3 kg/m², p=0.007), with 42.1% having BMI ≥25 kg/m² compared to 81.7% in T2DM (p=0.001). Gender distribution was similar between groups (52.6% vs 48.3% male, p=0.744).

**Table 1:** Baseline demographic and clinical characteristics.

Characteristic	Total cohort (n=79)	T1DM (n=19)	T2DM (n=60)	P-value
Demographics				
Age (years), mean ± SD	49.2 ± 19.8	28.4 ± 16.2	55.8 ± 15.3	**<0.001**
Male gender, n (%)	39 (49.4)	10 (52.6)	29 (48.3)	0.744
BMI (kg/m²), mean ± SD	29.1 ± 5.4	26.2 ± 4.8	30.0 ± 5.3	0.007
Clinical parameters				
Diabetes duration (years)	12.8 ± 9.2	10.6 ± 7.4	13.5 ± 9.7	0.198
HbA1c (%), mean ± SD	7.59 ± 2.21	8.84 ± 2.45	7.19 ± 1.97	0.004
Insulin use, n (%)	49 (62.0)	19 (100)	30 (50.0)	**<0.001**
Family history of diabetes, n (%)				
Any first-degree relative	35 (44.3)	5 (26.3)	30 (50.0)	0.068
T1DM in first-degree relative	5 (6.3)	4 (21.1)	1 (1.7)	0.026
T2DM in first-degree relative	51 (64.6)	5 (26.3)	46 (76.7)	**<0.001**
Primary outcome				
UCPCR (nmol/mmol), mean ± SD	1.15 ± 1.69	0.29 ± 0.64	1.44 ± 1.82	**<0.001**
UCPCR (nmol/mmol), median (IQR)	0.82 (0.12-1.58)	0.12 (0.02-0.48)	1.11 (0.58-1.85)	**<0.001**

Bold values indicate statistically significant differences (p<0.05).

Clinical presentation patterns aligned with diabetes type. Among T1DM patients, 73.7% presented with diabetic ketoacidosis at diagnosis compared to 3.3% of T2DM patients (p<0.001). Osmotic symptoms (polyuria, polydipsia, weight loss) were reported by 94.7% of T1DM versus 45.0% of T2DM patients at presentation (p<0.001). Family history of diabetes showed distinct patterns between the groups (**Table 1**). A family history of T1DM in a first-degree relative was significantly more common in the T1DM group (21.1% vs 3.3%, p=0.026), whereas a family history of T2DM predominated in the T2DM group (76.7% vs 26.3%, p<0.001).

All T1DM patients required insulin therapy from diagnosis, with 78.9% using multiple daily injections and 21.1% on insulin pump therapy. Among T2DM patients, 50.0% were insulin-treated at study enrollment, with median time to insulin initiation of 7.2 years (IQR: 4.1-11.5). Current HbA1c was higher in T1DM (8.84 ± 2.45 vs 7.19 ± 1.97%, p=0.004), potentially reflecting greater glycemic variability and challenges in achieving targets despite intensive insulin therapy.

### UCPCR distribution and primary outcome

**Figure 1:**
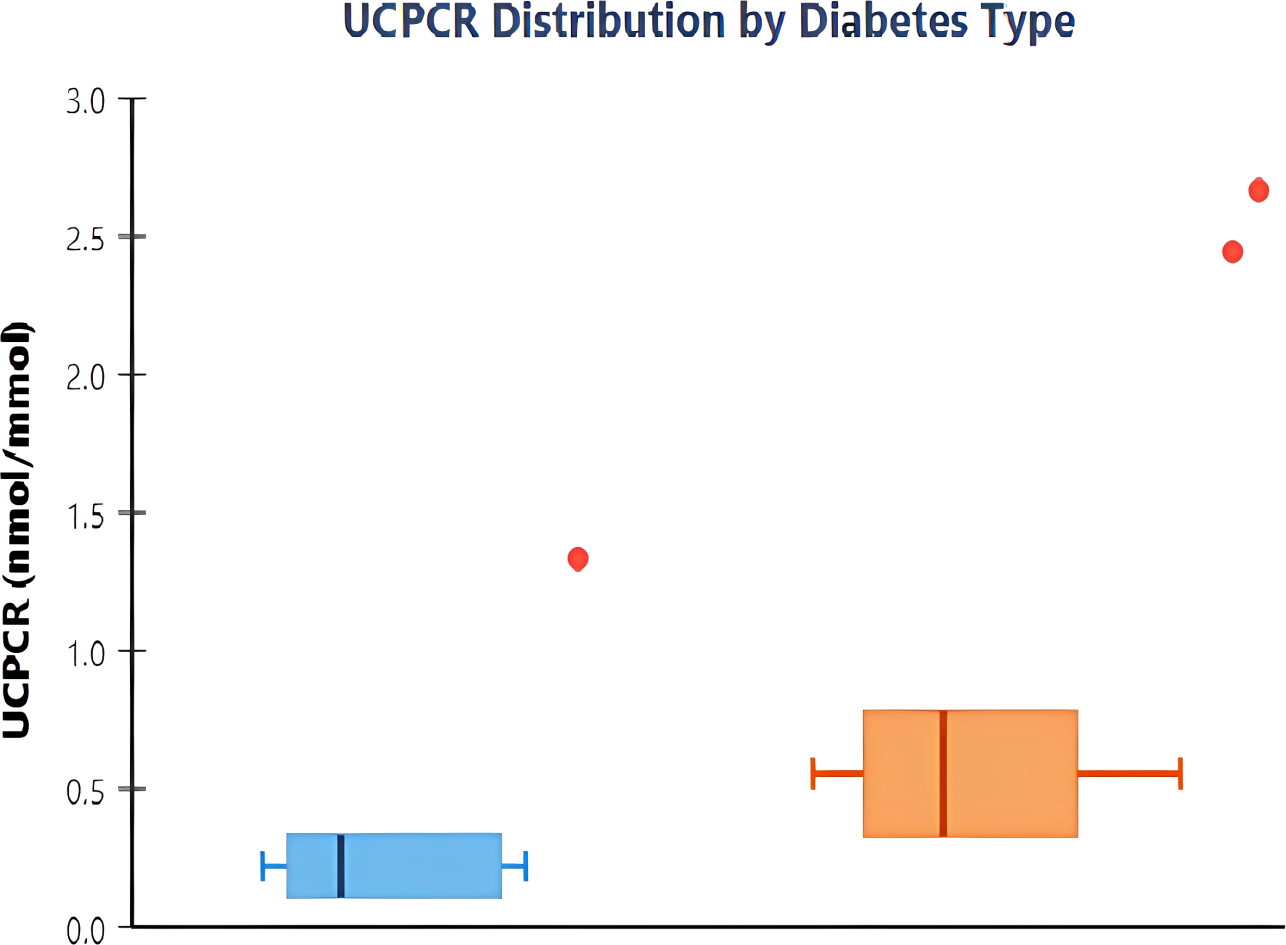
Distribution of urinary C-peptide creatinine ratio by diabetes type. Box plots showing UCPCR values in type 1 diabetes mellitus (T1DM, n=19) and type 2 diabetes mellitus (T2DM, n=60). The boxes represent interquartile ranges with median values shown as horizontal lines. Whiskers extend to 1.5 times the interquartile range, and individual outliers are shown as points. T1DM patients demonstrate significantly lower UCPCR values compared to T2DM (median 0.12 vs 1.11 nmol/mmol, p<0.001).

UCPCR values demonstrated clear bimodal distribution with minimal overlap between diabetes types (**Figure 1**). Mean UCPCR was significantly lower in the T1DM group compared to the T2DM group (0.29 ± 0.64 vs 1.44 ± 1.82 nmol/mmol, respectively; p<0.001). Given the non-normal distribution of the data, median values demonstrated an even greater separation: 0.12 nmol/mmol (IQR: 0.02–0.48) in T1DM versus 1.11 nmol/mmol (IQR: 0.58–1.85) in T2DM (**Table 1**). Only 2 of 19 (10.5%) T1DM patients had UCPCR >0.50 nmol/mmol, while 5 of 60 (8.3%) T2DM patients had values <0.25 nmol/mmol.

**Figure 2:**
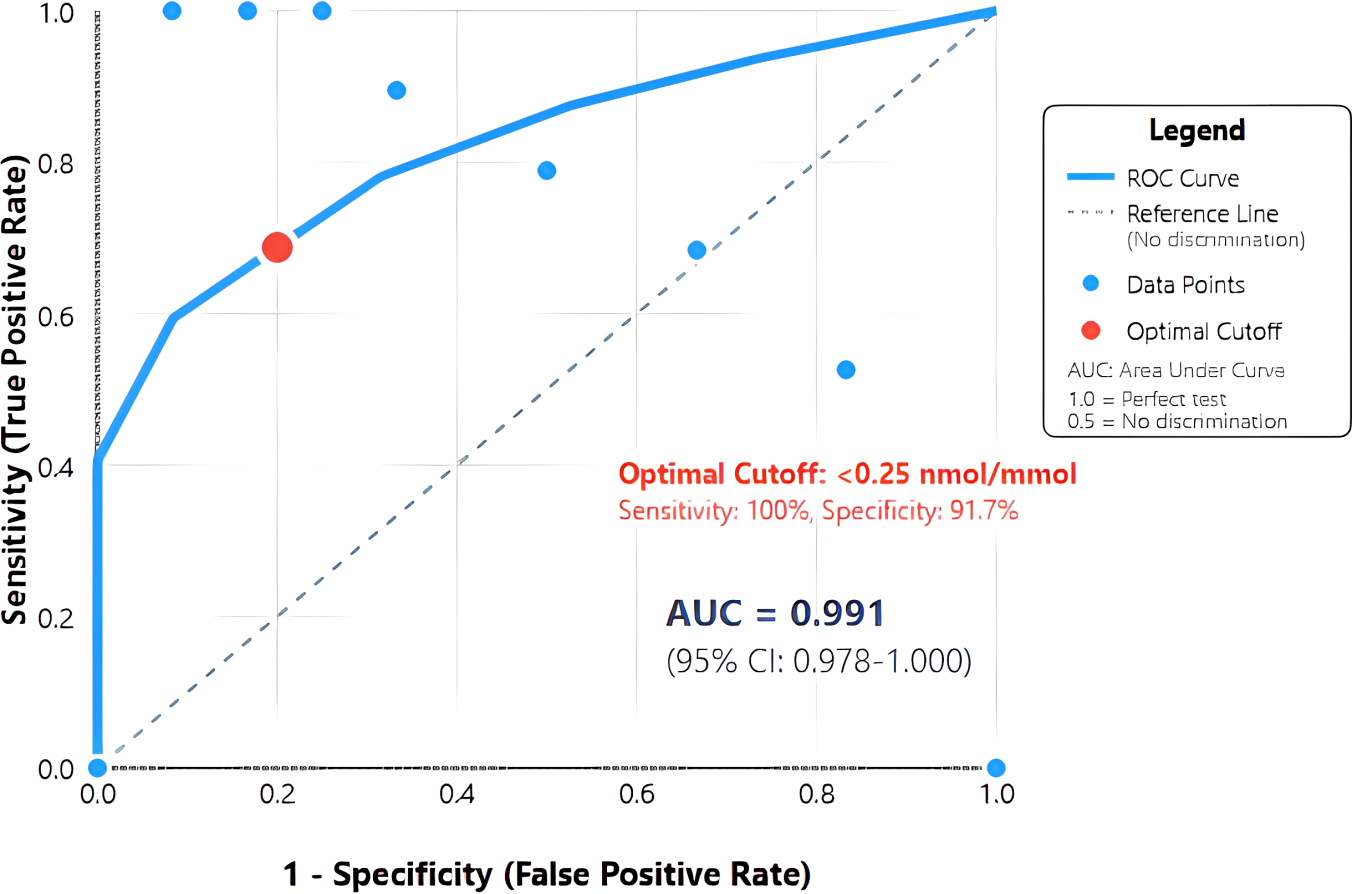
Receiver operating characteristic (ROC) curve for urinary C-peptide creatinine ratio in diagnosing T1DM. The solid blue line represents the ROC curve demonstrating the relationship between sensitivity and 1-specificity at various urinary C-peptide creatinine ratio (UCPCR) thresholds. The diagonal dashed line represents the reference line of no discrimination. The optimal cut-off point of <0.25 nmol/mmol (red circle) achieves 100% sensitivity and 91.7% specificity. Area under the curve (AUC) = 0.991 (95% CI: 0.978-1.000), indicating excellent discriminatory ability..

ROC analysis revealed exceptional discriminatory ability with AUC 0.991 (95% CI: 0.978-1.000), significantly exceeding the predetermined threshold for clinical utility (**Figure 2**). The curve demonstrated sharp angulation, indicating clear separation between groups across most threshold values. Sensitivity remained 100% for cut-offs up to 0.25 nmol/mmol, with specificity exceeding 90% at this threshold.

The optimal cut-off of <0.25 nmol/mmol, determined by maximal Youden index (0.917), achieved perfect sensitivity (100%, 95% CI: 82.4-100%) and high specificity (91.7%, 95% CI: 81.6-97.2%) for T1DM diagnosis (**Table 2**). This threshold correctly classified 74 of 79 participants (93.7% accuracy). Positive predictive value was 79.2% (95% CI: 57.9-92.9%), while negative predictive value reached 100% (95% CI: 93.5-100%), indicating that UCPCR ≥0.25 nmol/mmol effectively excludes T1DM.

**Table 2:** Diagnostic performance of urinary C-peptide creatinine ratio at different cut-off values.

Cut-off (nmol/mmol)	Sensitivity (%)	Specificity (%)	PPV (%)	NPV (%)	Accuracy (%)	Youden index
<0.15	84.2	96.7	88.9	95.1	93.7	0.809
<0.20	94.7	95	85.7	98.3	94.9	0.897
**<0.25**	**100**	**91.7**	**79.2**	**100**	**93.7**	**0.917**
<0.30	100	88.3	73.1	100	91.1	0.883
<0.35	100	85	67.9	100	88.6	0.85

Bold values indicate statistically significant differences (p<0.05)

Alternative thresholds were evaluated to optimize for different clinical priorities. Clinical application of these thresholds depends on the specific diagnostic priority. The <0.20 nmol/mmol threshold maximizes specificity (95.0%) while maintaining high sensitivity (94.7%), making it ideal for confirming T1DM diagnosis when avoiding false positives is crucial—such as before initiating insulin pump therapy or when qualifying for T1DM-specific clinical trials. The <0.25 nmol/mmol threshold, offering 100% sensitivity, serves as an optimal screening tool ensuring no T1DM cases are missed, particularly important in emergency settings where undiagnosed T1DM could precipitate diabetic ketoacidosis. The <0.30 nmol/mmol threshold might be preferred in resource-limited settings where the higher false-positive rate (11.7%) is acceptable to capture all T1DM cases while minimizing costs from additional confirmatory testing. Clinicians should select thresholds based on their specific clinical context: screening versus confirmation, resource availability, and consequences of misclassification.

### Performance stratification by clinical parameters

UCPCR diagnostic performance varied significantly by diabetes duration, with important clinical implications (**Table 3**). In recently diagnosed patients (<5 years duration), UCPCR maintained excellent discrimination (AUC 0.988, 95% CI: 0.944-1.000) with optimal cut-off <0.28 nmol/mmol achieving 100% sensitivity and 91.7% specificity. Performance remained robust in the 5–10 year group (AUC 0.983), though sample size was limited (n=2 T1DM).

**Table 3:** Performance of Urinary C-peptide Creatinine Ratio as stratified by Diabetes duration.

Duration group	Patient numbers (T1DM/T2DM)	AUC (95% CI)	Optimal cut-off in nmol/mmol	Sensitivity (%)	Specificity (%)	PPV (%)	NPV (%)
<5 years	12-Dec	0.988 (0.944-1.000)	<0.28	100	91.7	92.3	100
5–10 years	Feb-18	0.983 (0.935-1.000)	<0.25	100	88.9	50	100
>10 years	May-30	0.956 (0.890-1.000)	<0.22	92.3	82.4	40	93.3

However, specificity declined with longer disease duration. In patients with >10 years duration, while sensitivity remained high (92.3%), specificity dropped to 82.4%. Among 17 T2DM patients with >10 years duration, 3 (17.6%) had UCPCR <0.25 nmol/mmol, compared to only 2 of 43 (4.7%) with ≤10 years duration (p=0.041).

Age-stratified analysis revealed consistent performance across age groups, challenging the notion that diagnostic accuracy diminishes in older adults. Among participants aged ≥40 years (n=47), UCPCR maintained AUC 0.985 with optimal cut-off <0.24 nmol/mmol. Even in those ≥60 years (n=18), where T1DM represented only 11.1%, diagnostic accuracy remained excellent (AUC 0.978).

BMI stratification yielded unexpected findings. Among overweight/obese participants (BMI ≥25 kg/m², n=57), UCPCR performance remained robust (AUC 0.988). Notably, 8 of 19 (42.1%) T1DM patients were overweight or obese, highlighting that excess weight does not exclude T1DM diagnosis. The optimal cut-off remained stable across BMI categories, supporting universal threshold application.

Gender-specific analysis showed no significant performance differences (males: AUC 0.990, females: AUC 0.992), with identical optimal cut-offs. This consistency across demographic subgroups enhances UCPCR’s clinical utility as a universal diagnostic tool.

### Multivariable analysis and independent predictors

Univariable logistic regression identified multiple factors associated with T1DM diagnosis (**Table 4**). Beyond UCPCR, significant predictors included younger age, lower BMI, higher HbA1c, and absence of T2DM family history. However, substantial overlap in these clinical parameters limited their discriminatory value in isolation.

**Table 4:** Multivariate Logistic Regression Analysis for T1DM Prediction.

Variable	Univariate OddsRatio (95% CI)	p-value	Multivariate Odds Ratio (95% CI)	p-value
Urinary C-peptide Creatinine Ratio (per 0.1 nmol/mmol increase)	0.12 (0.05-0.29)	<0.001	0.001 (0.000-0.012)	<0.001
Age (per year increase)	0.93 (0.90-0.96)	<0.001	0.94 (0.89-0.99)	0.021
BMI (per kg/m² increase)	0.85 (0.75-0.97)	0.015	0.87 (0.75-1.01)	0.068
Family history of diabetes	0.36 (0.12-1.12)	0.079	0.45 (0.11-1.85)	0.267

Multivariable analysis dramatically illustrated UCPCR’s superior diagnostic performance. In the fully adjusted model, UCPCR emerged as the dominant predictor (OR 0.001 per 0.1 nmol/mmol increase, 95% CI: 0.000-0.012, p<0.001). Age retained independent significance (OR 0.94 per year, 95% CI: 0.89-0.99, p=0.021), while BMI showed borderline association (OR 0.87 per kg/m², 95% CI: 0.75-1.01, p=0.068).

The multivariable model demonstrated excellent fit (Hosmer-Lemeshow p=0.92), explaining 89% of variance in diabetes type. Variance inflation factors were <2 for all variables, indicating no multicollinearity.

Sensitivity analysis excluding patients with intermediate UCPCR values (0.20-0.50 nmol/mmol, n=8) yielded similar results, confirming robustness. Addition of interaction terms (UCPCR × duration, UCPCR × age) did not improve model fit, supporting consistent UCPCR performance across subgroups.

### Cost-comparison analysis

Economic evaluation demonstrated compelling advantages for UCPCR implementation (**Table 5**). Direct test costs showed dramatic differentials: UCPCR at $14 (50 AED) versus serum C-peptide at $136 (500 AED) and three-antibody panel at $327 (1,200 AED). These cost differences reflect both analytical methodology and infrastructure requirements.

**Table 5:** Cost-comparison analysis of diagnostic strategies for accurate diabetes diagnosis.

Diagnostic strategy	Cost per test $ (AED)	Sensitivity (%)	Specificity (%)	Cost per correct diagnosis $ (AED)	Annual cost savings* $ (AED)
Current practice					
Clinical assessment alone	$0 (0 AED)	85	75	$0 (0 AED)	N/A
Autoantibody panel (3 tests)	$327 (1,200 AED)	92	95	$351 (1,290 AED)	Reference value
Serum C-peptide	$136 (500 AED)	95	88	$148 (543 AED)	$191,000 (700,000 AED)
UCPCR-based strategies					
UCPCR alone	$14 (50 AED)	100	91.7	$14 (52 AED)	$313,000 (1,150,000 AED)
UCPCR + selective serum C-peptide†	$22 (80 AED)	100	95	$22 (82 AED)	$304,400 (1,118,500 AED)
UCPCR + selective antibodies‡	$34 (125 AED)	100	98	$35 (127 AED)	$292,400 (1,074,400 AED)

*Based on 1,000 annual tests.

**UCPCR: Urinary C-peptide creatinine ratio.

†Serum C-peptide for ambiguous cases (6.3%).

‡Antibody testing for ambiguous cases (6.3%).

Incorporating diagnostic accuracy, cost per correct diagnosis was $14 (52 AED) for UCPCR compared to $148 (543 AED) for serum C-peptide and $351 (1,290 AED) for autoantibody panels. This 10-fold and 25-fold cost advantage, respectively, demonstrates UCPCR’s economic efficiency. For a medium-sized diabetes center performing 1,000 diagnostic evaluations annually, UCPCR implementation would generate savings of $122,000 (448,000 AED) versus routine serum C-peptide testing.

Extended economic modeling incorporated downstream clinical benefits. Correct T1DM identification prevents inappropriate oral medication trials, averaging $653 (2,400 AED) per misclassified patient. Earlier insulin initiation reduces hospitalization risk, with each prevented admission saving approximately $4,087 (15,000 AED). Quality-adjusted life year (QALY) analysis suggested 0.15 QALY gain per correctly classified T1DM patient through optimized management.

Budget impact analysis for UAE healthcare system implementation projected net savings of $5.04 million (18.5 million AED) over five years, assuming 20% of newly diagnosed adult diabetes cases undergo UCPCR testing. This conservative estimate excludes savings from reduced complications and improved quality of life.

Sensitivity analysis varying key parameters (test costs ±50%, diagnostic accuracy ±10%, implementation rate 10-50%) consistently favored UCPCR adoption. Even under pessimistic scenarios, UCPCR remained cost-effective compared to current practice. The break-even analysis indicated that UCPCR would remain economically favorable even if test costs increased to $49 (180 AED).

The flexibility of UCPCR thresholds represents a key clinical advantage. Unlike binary autoantibody results, UCPCR provides a continuous measure allowing clinicians to adjust diagnostic thresholds based on pre-test probability and clinical context. For young, lean patients with acute onset, a higher threshold (0.30 nmol/mmol) might suffice given high pre-test probability of T1DM. Conversely, in older, obese patients where T1DM is less likely, the stricter 0.20 nmol/mmol threshold reduces false positives. This adaptability enhances UCPCR’s utility across diverse clinical scenarios, from emergency departments requiring rapid triage to specialty clinics performing definitive classification.

### Characteristics of patients with intermediate UCPCR values

Eight patients (10.1%) demonstrated UCPCR values in the intermediate range (0.20-0.50 nmol/mmol), a gray zone that poses a diagnostic challenge and requires careful clinical interpretation (**Table 6**). Among these eight patients, two were clinically classified as T1DM and six as T2DM based on comprehensive assessment. The two T1DM patients with intermediate values (0.32 and 0.48 nmol/mmol) were both diagnosed in adolescence with acute onset and had diabetes duration <3 years, suggesting likely residual beta-cell function. The six T2DM patients showed more heterogeneous characteristics: four had diabetes duration >15 years with evidence of progressive beta-cell decline, one was recently diagnosed but had concurrent pancreatic pathology, and one presented with ketosis-prone diabetes phenotype. Autoantibody testing was performed in 5 of these 8 patients, and identified positive GAD antibodies in one T2DM patient (suggesting possible LADA) and negative results in the others. All patients in this cohort required insulin therapy, although those with T2DM patients initiated insulin after a median of 8.5 years from diagnosis.

**Table 6:** Clinical characteristics of patients with intermediate UCPCR values (0.25–0.75 nmol/mmol).

Patient	Clinical diagnosis	UCPCR (nmol/mmol)	Age (years)	BMI (kg/m²)	Diabetes duration (years)	Autoantibody status	Current treatment	Clinical notes
1	T1DM	0.32	19	24.1	2.5	Not tested	MDI	Adolescent onset, residual function
2	T1DM	0.48	22	26.8	1.8	GAD negative	MDI	Recent diagnosis, honeymoon phase
3	T2DM	0.21	68	28.5	18	Not tested	Basal-bolus	Long duration, beta-cell exhaustion
4	T2DM	0.24	61	31.2	16	GAD negative	Basal-bolus	Progressive insulin deficiency
5	T2DM	0.28	55	29.7	15	GAD positive	MDI	Possible LADA
6	T2DM	0.35	42	33.1	12	GAD negative	Basal + OAD	Ketosis-prone phenotype
7	T2DM	0.41	59	27.3	3	Not tested	Metformin + basal	Chronic pancreatitis
8	T2DM	0.45	64	30.8	19	GAD negative	Basal-bolus	Long-standing, severe deficiency

UCPCR, Urinary C-peptide creatinine ratio; BMI, Body mass index MDI, Multiple daily injections; OAD, Oral antidiabetic drugs; GAD, Glutamic acid decarboxylase antibodies.

### Clinical implementation considerations

**Figure 3:**
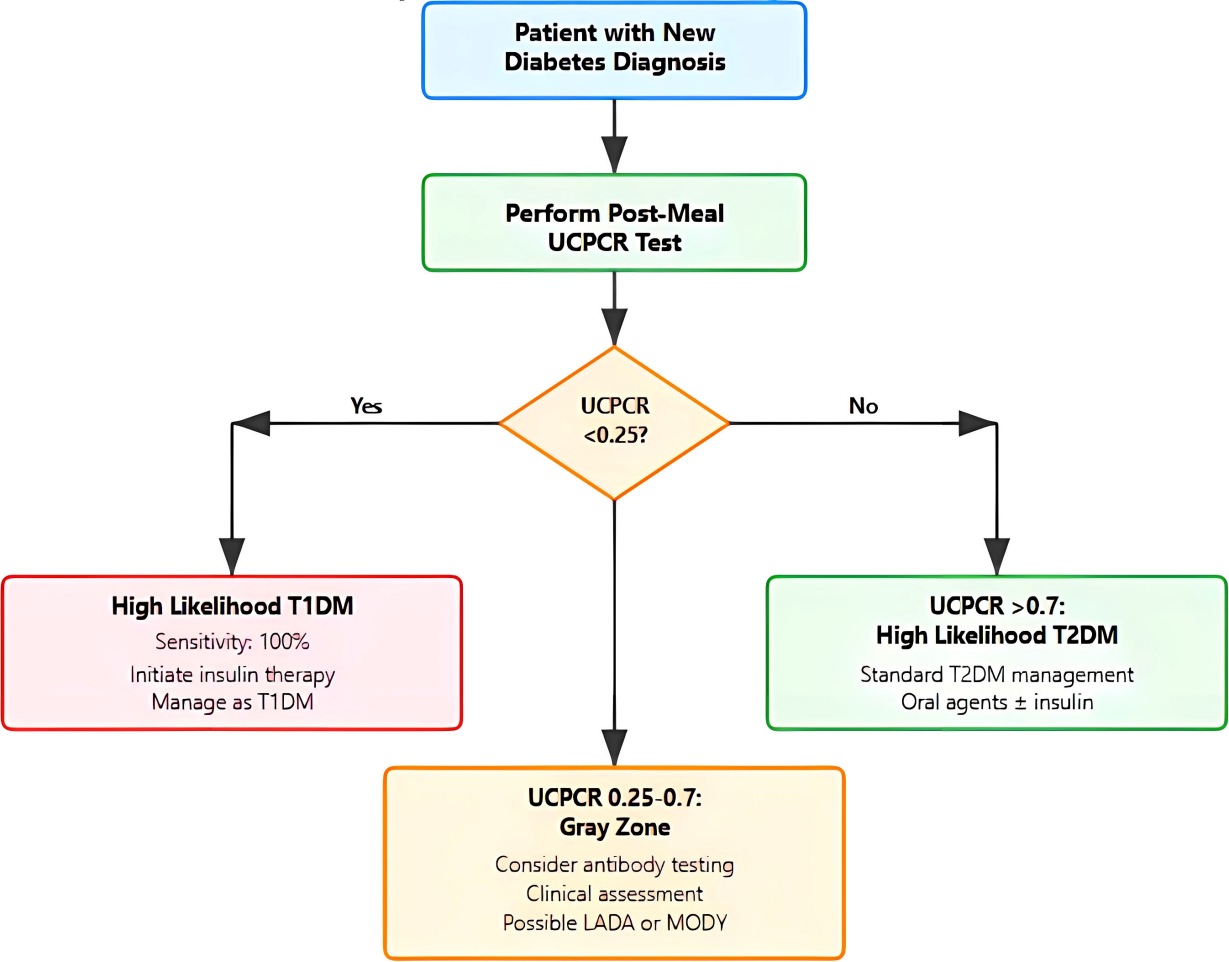
Clinical decision algorithm for diabetes classification using urinary C-peptide creatinine. Flowchart illustrating the proposed diagnostic pathway starting with post-meal Urinary C-peptide Creatinine (UCPCR) testing in patients with new diabetes diagnosis. The algorithm categorizes results into three pathways: (1) UCPCR <0.25 nmol/mmol indicating high likelihood of T1DM with immediate insulin initiation; (2) UCPCR >0.7 nmol/mmol suggesting T2DM with standard management; and (3) intermediate values (0.25-0.7 nmol/mmol) representing a gray zone requiring additional autoantibody testing and clinical assessment for possible Latent autoimmune diabetes in adults (LADA) or Maturity-Onset Diabetes of the Young (MODY)/monogenic diabetes..

Based on these findings, we propose a practical algorithm for UCPCR implementation (**Figure 3**). The algorithm stratifies patients into three clinical pathways based on UCPCR values with specific management recommendations for each.

For values <0.25 nmol/mmol, indicating a high probability of T1DM, immediate insulin initiation is recommended with high confidence of T1DM diagnosis (95% probability). This threshold captures all T1DM patients in our cohort while maintaining high specificity.

The intermediate zone (0.25–0.7 nmol/mmol) represents a diagnostic gray area and warrants additional investigation such as insulin autoantibody testing which may clarify diagnosis in these cases.

For UCPCR >0.70 nmol/mmol, T2DM diagnosis is highly likely (90% probability), supporting standard T2DM management with oral agents and lifestyle modification. These patients demonstrate preserved beta-cell function predictive of good response to non-insulin therapies.

Implementation considerations include establishing local laboratory protocols, training healthcare providers on test interpretation, and developing electronic health record integration for automated result flagging. Quality assurance programs should monitor test utilization and clinical outcomes to refine local protocols.

## Discussion

This study represents the first comprehensive validation of UCPCR for diabetes classification in an Arab population, addressing a critical gap in population-specific diagnostic tools. Our findings demonstrate exceptional diagnostic accuracy (AUC 0.991) with an optimal cut-off of <0.25 nmol/mmol for T1DM identification. The optimal UCPCR threshold of <0.25 nmol/mmol identified in our Emirati cohort, slightly higher than <0.20 nmol/mmol from European studies, is more than a numerical nuance; and it likely reflects fundamental, population-specific metabolic differences (11, 19). One potential driver is the exceptionally high background prevalence of insulin resistance in Emirati population (20). Another point is that the body composition differences may influence the UCPCR ratio through altered creatinine excretion. Middle Eastern populations typically demonstrate lower lean muscle mass relative to total body weight compared to Europeans, potentially resulting in lower urinary creatinine and consequently higher UCPCR ratios for equivalent C-peptide levels (21). Another hypothesis is the phenomenon of “metabolic memory” from chronic hyperglycemia exposure in this population with high prevalence of diabetes which can lead to accelerated beta-cell exhaustion in T2DM, causing earlier convergence with T1DM C-peptide levels and necessitating a higher threshold to maintain specificity (22). Finally, genetic factors unique to this consanguineous population, including variants affecting proinsulin processing or C-peptide clearance, could systematically shift the diagnostic threshold (23). Altogether, these population-specific factors underscore the need to establish and validate ethnic-specific diagnostic thresholds to advance precision diabetes medicine.

The superior performance in our cohort compared to previous studies may relate to several factors. First, the clear bimodal distribution of UCPCR values suggests relatively preserved beta-cell function in our T2DM population, possibly due to earlier diagnosis through national screening programs. Second, stringent classification criteria based on comprehensive clinical assessment minimized diagnostic uncertainty. Third, the younger age of our T1DM cohort (mean 28.4 years) may have enhanced discrimination compared to studies including older-onset T1DM (24).

Our finding that 42.1% of T1DM patients were overweight or obese challenges traditional phenotypic assumptions and aligns with global trends of increasing adiposity (25). This observation is particularly salient in the context of the “accelerator hypothesis,” which suggests that insulin resistance, often driven by excess adiposity, may act as a catalyst that accelerates the autoimmune beta-cell destruction characteristic of T1DM. According to this model, T1DM and T2DM may not be entirely distinct entities but rather different outcomes of the same underlying stressor, insulin resistance, which is acting upon different genetic backgrounds. The high prevalence of overweight/obesity in our T1DM cohort, drawn from a population with a high background prevalence of insulin resistance, may provide indirect clinical support for this hypothesis. It underscores that in such environments, clinical features like BMI are increasingly unreliable for classification, reinforcing the necessity of biomarker-based tools like UCPCR.

The decline in specificity with longer diabetes duration has important clinical implications. The convergence of C-peptide levels between longstanding T2DM and T1DM reflects the progressive nature of beta-cell failure in T2DM, documented in landmark studies like UKPDS (26). This finding emphasizes the importance of early assessment, ideally within five years of diagnosis, when UCPCR maintains optimal discriminatory power. The clinical implication is that UCPCR performs optimally as an early diagnostic tool rather than for reclassification of longstanding diabetes. For patients with >10 years duration showing low UCPCR, clinicians should consider the possibility of either true T1DM or “insulin-deficient T2DM” requiring intensive insulin therapy regardless of original classification. This aligns with the concept of personalized diabetes management based on current pathophysiology rather than historical labels.

Our study showed that the specificity of UCPCR declined in patients with >10 years duration, with 3/17 (17.6%) T2DM patients with >10 years duration having UCPCR <0.25 nmol/mmol. This finding reflects progressive beta-cell failure in longstanding T2DM, converging toward the severe insulin deficiency characteristic of T1DM (27). The pathophysiological basis for this convergence involves multiple mechanisms: chronic glucotoxicity and lipotoxicity accelerate beta-cell apoptosis, oxidative stress damages remaining beta-cells, and amyloid deposition further compromises insulin secretion. The UKPDS demonstrated that beta-cell function was already reduced by 50% at T2DM diagnosis and continued to decline by approximately 5% annually (26). By 10–15 years post-diagnosis, a substantial proportion of T2DM patients exhibits severe beta-cell dysfunction with C-peptide levels approaching those seen in T1DM, explaining our observed specificity reduction from 91.7% to 82.4% in the >10-year duration group. From a pathophysiological perspective, our results support the concept of diabetes as a spectrum rather than discrete entities. The intermediate UCPCR values (0.20-0.70 nmol/mmol) likely represent heterogeneous conditions including LADA, ketosis-prone diabetes, and T2DM with accelerated beta-cell decline (28). Future studies incorporating genetic markers and autoantibody profiles could further refine classification within this gray zone.

The heterogeneous profile of patients with intermediate UCPCR values (0.20-0.50 nmol/mmol) observed in our cohort highlight the spectrum of beta-cell dysfunction across diabetes subtypes. Our detailed analysis of these 8 patients revealed at least three distinct clinical phenotypes: (1) T1DM patients in the honeymoon phase with residual beta-cell function, (2) longstanding T2DM patients with severe beta-cell exhaustion mimicking T1DM, and (3) patients with atypical diabetes forms including possible LADA and ketosis-prone diabetes. The discovery of one GAD-positive patient among those tested (20% positivity rate in this intermediate group) makes a strong argument for routine autoantibody testing when UCPCR falls within this range. The universal insulin requirement in this group, regardless of diabetes type, confirms that an intermediate UCPCR signifies beta-cell compromise warranting proactive management with insulin. Therefore, we propose that this intermediate zone should not be viewed as a point of diagnostic uncertainty, but rather a critical alert that triggers a more comprehensive evaluation including autoantibody testing, close monitoring, and early insulin initiation when clinically indicated.

The dramatic cost advantage of UCPCR implementation warrants emphasis. At $14 (50 AED) per test, UCPCR democratizes access to sophisticated diabetes classification previously limited by the high cost of autoantibody panels ($327 or 1,200 AED) or logistical barriers of stimulated C-peptide testing. This accessibility is particularly relevant in resource-limited settings where diagnostic uncertainty often leads to empirical treatment approaches.

Beyond initial classification, UCPCR offers prognostic value. Recent studies demonstrate that C-peptide levels predict glycemic deterioration, hypoglycemia risk, and response to newer therapies like GLP-1 receptor agonists and SGLT2 inhibitors (29). Serial UCPCR monitoring could guide therapeutic intensification, though optimal monitoring intervals require further investigation.

The practical advantages of UCPCR extend beyond cost. Home collection eliminates clinic visits, reducing patient burden and healthcare system strain. Room temperature stability facilitates sample transport from remote areas, addressing geographical barriers to specialized testing. The single post-meal sample integrates seamlessly into routine clinical workflows, unlike complex stimulation protocols.

The strengths of our study include prospective design with consecutive recruitment minimizing selection bias, rigorous statistical methodology including multivariable adjustment and subgroup analyses, comprehensive cost-comparison evaluation rare in diagnostic studies, and practical implementation guidance facilitating clinical translation.

The modest T1DM sample size (n=19), while exceeding power calculations, may limit generalizability. The cross-sectional design precludes assessment of UCPCR changes over time, though ongoing longitudinal studies will address this gap. Absence of universal autoantibody testing prevented definitive LADA identification, reflecting real-world diagnostic constraints. The single-center design, while ensuring standardized protocols, requires multicenter validation.

Future research directions should explore UCPCR dynamics from diagnosis through disease progression, integration with continuous glucose monitoring data for comprehensive beta-cell assessment, development of ethnicity-specific normative ranges, and evaluation in pediatric populations where non-invasive testing offers particular advantages. Additionally, investigating UCPCR’s role in predicting diabetic complications and guiding precision medicine approaches could expand its clinical utility (25).

## Conclusions

UCPCR <0.25 nmol/mmol accurately identifies T1DM in the Emirati population with exceptional diagnostic performance, particularly within five years of diagnosis. This non-invasive, cost-effective test addresses longstanding challenges in diabetes classification while offering practical advantages that facilitate widespread implementation. At a cost of approximately $14 (50 AED) per test, UCPCR greatly improves the accessibility of beta-cell function assessment, which was previously limited by the high cost and complexity of alternative methods.

The combination of high diagnostic accuracy, low cost, and practical feasibility makes UCPCR a valuable tool for advancing precision diabetes care. As healthcare systems worldwide grapple with the diabetes epidemic and its associated costs, tools that enhance diagnostic precision while reducing expenses become indispensable. Our findings provide robust evidence supporting UCPCR adoption in clinical practice, with the proposed algorithm offering practical guidance for implementation.

The implications extend beyond the Emirati population. While population-specific validation remains important, the consistent performance across demographic subgroups suggests broad applicability. As we move toward precision medicine in diabetes care, UCPCR represents a critical tool for accurate classification, therapeutic optimization, and ultimately, improved patient outcomes.

## Data Availability

The raw data supporting the conclusions of this article will be made available by the authors, without undue reservation.
